# Research Software vs. Research Data II: Protocols for Research Data dissemination and evaluation in the Open Science context

**DOI:** 10.12688/f1000research.78459.1

**Published:** 2022-01-28

**Authors:** Teresa Gomez-Diaz, Tomas Recio

**Affiliations:** 1 Laboratoire d'informatique Gaspard-Monge, CNRS, Paris-Est, France; 2 Universidad Antonio de Nebrija, Madrid, Spain

**Keywords:** Research Data, Research Software, Open Science, Research outputs’ dissemination, Research Evaluation, FAIR principles.

## Abstract

**Background: **Open Science seeks to render research outputs visible, accessible and reusable. In this context, Research Data and Research Software sharing and dissemination issues provide real challenges to the scientific community, as consequence of recent progress in political, legal and funding requirements.

**Methods: **We take advantage from the approach we have developed in a precedent publication, in which we have highlighted the similarities between the Research Data and Research Software definitions.

**Results:** The similarities between Research Data and Research Software definitions can be extended to propose protocols for Research Data dissemination and evaluation derived from those already proposed for Research Software dissemination and evaluation. We also analyze FAIR principles for these outputs.

**Conclusions:** Our proposals here provide concrete instructions for Research Data and Research Software producers to make them more findable and accessible, as well as arguments to choose suitable dissemination platforms to complete the FAIR framework. Future work could analyze the potential extension of this parallelism to other kinds of research outputs that are disseminated under similar conditions to those of Research Data and Research Software, that is, without widely accepted publication procedures involving editors or other external actors and where the dissemination is usually restricted through the hands of the production team.

## 1. Introduction

Researchers produce many different outputs in their work in order to obtain the results that will be published in scientific journals, in articles that are still the main exchanging information mechanism in the scientific conversation. Among others, researchers produce Research Data (RD) and Research Software (RS), but yet again, both outputs have not currently a publication procedure as widely accepted as the one existing for articles, which constitutes one of the main drawbacks for their acceptance as *first class citizens* in the scientific ecosystem. This is one of the goals of the FAIR guiding principles 1:

*…is for scholarly digital objects of all kinds to become ‘first class citizens’ in the scientific publication ecosystem, where the quality of the publication – and more importantly, the impact of the publication – is a function of its ability to be accurately and appropriately found, reused, and cited over time, by all stakeholders, both human and mechanical.*

*[…] we do not pay our valuable digital objects the careful attention they deserve when we create and preserve them.*

On the other hand, the following definition sets up Open Science goals related to research outputs 2:

*Open Science is the political and legal framework where research outputs are shared and disseminated in order to be rendered visible, accessible and reusable.*

In this context, as reported in 3, the necessary skills to reach out these goals are complex:

*The skills needed for Open Science cover a broad span from data management to legal aspects, and include also more technical skills, such as data stewardship, data protection, scholarly communication and dissemination (including creating metadata)…*

and still require to be engineered 4 (see also section 5 of 5):

*An acceptable workflow needs to be created. However, most researchers, while experts in their own fields, have little awareness of metadata standards for data publication and information science in general, leading to cognitive and skill barriers that prevent them from undertaking routine best-practice data management.*

Another drawback of this missing publication procedure for RD and RS is the possible loss of the expert knowledge that has been acquired along the research process 6:

*If not traditional papers and volumes, what, then, should researchers be publishing? Whilst the digital exchange of data is straightforward, the digital exchange and transfer of scientific knowledge in collaborative environments has proven to be a non-trivial task, requiring tacit, and rapidly changing expert knowledge – much of which is lost in traditional methods of publication and information exchange. We believe that there is a need for mechanisms that support the production of self-contained units of knowledge and that facilitate the publication, sharing and reuse of such entities.*

Examples of this lost knowledge include the report of failure cases, which are rarely published; or the description of the modifications that have been included in the final implemented algorithms, and that are the result of a long trial and error process to improve the initially conceived algorithm or to avoid computational errors.

Although the current trend in the scientific publication ecosystem is to place RS and RD into a better position, many researchers are still at a loss when facing RS and RD dissemination, and do not possess the needed skills, support or assistance for their disclosure in the right conditions. Moreover, they consider that much work and effort would be necessary to accomplish this goal, while having little or no positive effect in their curriculum 4:

*Put crudely, the large amount of effort involved in preparing data for publication release, coupled with the negligible current incentives and rewards, prevents many researchers from doing so.*

On the other hand, research funders, like the European Commission, are currently laying out Open Science policies in their calls, in which it is required open access to the generated RD of the funded projects (although there may be exceptions), and where it is recommended to provide open access to research outputs in all generality, beyond publications and data, e.g. software tools 30. Notice that in the dissemination of these research outputs it is necessary to provide significant information in order to facilitate their visibility, accessibility and their reuse 7:

*Detailed provenance includes facets such as how the resource was generated, why it was generated, by whom, under what conditions, using what starting-data or source-resource, using what funding/resources, who owns the data, who should be given credit, and any filters or cleansing processes that have been applied post-generation.*

Bearing in mind the above described landscape, the goal of our work here is to contribute to the improvement of the scientific endeavor with protocols that could help researchers, and the community at large, in the dissemination of their produced RD and RS, while contributing to the accomplishment of Open Science goals.

We concentrate here in practical matters, that is, in the *how to*: how to disseminate RD and RS to make them *first class citizens* so that they become visible, accessible, reusable. But dissemination procedures are not enough. With the aim to motivate researchers to deal with better dissemination tasks, most of the times considered by the members of the scientific community as an additional, useless burden, we should also take into consideration pathways that yield improved research evaluation practices, so relevant for researchers. That is, pathways that contribute to evaluate correctly the disseminated outputs with protocols that help both the researchers – to know what will be evaluated and how – as well as the evaluators – into setting the evaluation process.

Our proposal is grounded on our knowledge and experience concerning RS 8- 12. This translation of knowledge from RS to RD has been already successfully applied 13 to propose a RD definition and to tackle some of the Borgman’s conundrum challenges 14. In the present paper we attempt to extend this approach to the case of RD dissemination and evaluation practices.

The plan of this work is as follows. The next section is devoted to revisit the corresponding points related to RS: definition, dissemination, evaluation and consideration of the role of FAIR principles in this context. Section 3 focus then in RD topics, reviewing the proposed RD definition 13 and to present the main contribution: some comprehensive RD dissemination and evaluation procedures. Conclusions will end this work.

## 2. Research Software

Three are the main components of this section: the RS definition coming from 11, 12, the RS dissemination procedure coming from 8, the CDUR RS evaluation protocol from 11. Some comments on FAIR principles for RS will complete this section.

### 2.1 Research Software definition, reference and citation

In this work we consider the following definition of RS 11, 12:

***Research software** is a well identified set of code that has been written by a (again, well identified) research team. It is software that has been built and used to produce a result published or disseminated in some article or scientific contribution. Each research software encloses a set (of files) that contains the source code and the compiled code. It can also include other elements as the documentation, specifications, use cases, a test suite, examples of input data and corresponding output data, and even preparatory material.*

We observe, following the above definition, that RS has three main characteristics:

• the goal of the RS development is to do research,
• it has been written by a research team,
• the RS is involved in the obtention of the results presented in scientific articles (as the most important means for scientific exchange are still articles published in scientific journals).

Note that documentation, licenses, examples, data, tests, software management plans and other related information and materials can also be part of the set of files that constitutes a specific RS.

Moreover, a RS development team may not just use software produced by other teams, but also include external software as a component inside the ongoing development, something which is facilitated by the Free/Open Source Software (FLOSS) ^1^ licenses. This potential external component will qualify here as RS if it complies with the three characteristics given in the above definition 13. Moreover, the responsible team of the resulting work should clearly identify the included external components and their licenses, as well as highlight, by means of recommended citation practices 8, 11, 15, the external components that qualify as RS.

General aspects of FLOSS issues can be consulted, for example, in 16. Let us remark that good practices for software development management ask for updating regularly the RS related information, like, for example, project’s funding, publications or involved teams and contributors. A Software Management Plan (SMP) can be a powerful tool to help and to handle this information, see for example 10.

Let us recall that RS reference and citation recommendations have been considered in section 2.5 of 11 where we propose easy to adopt methods to improve RS citation practices.

### 2.2 A Research Software dissemination procedure

Let us begin by recalling that, as stated in 30:

*Dissemination means the public disclosure of the results by appropriate means (other than resulting from protecting or exploiting the results), including by scientific publications in any medium.*

The following RS dissemination procedure has been proposed in 8 and was first published ^2^ in the PLUME project ^3^ (2006-2013) 11, 17. The French initial version includes a close analysis of legal issues (French author rights, licensing) in order to produce FLOSS RS. It is slightly updated and completed in the following. More information on the legal issues can be found in 9.

As a general recommendation, it is best practice to consider licensing issues and to keep them in a SMP from the very first stages of the RS development. The RS license establishes its sharing conditions: it gives rights for access, copy, modification, redistribution of the RS, and it can establish reciprocity clauses that should be respected by the potential RS users. Licenses should be put well into place before releasing the RS.

Here we present the proposed RS dissemination procedure. Steps marked with (*) are to be revisited regularly for each version release.

• Choose a name or title to identify the RS, avoid trademarks and other proprietary names, you can associate date, version number, and target platform. Consider best practices in file names ^4^.
• (*) Establish the list of authors and affiliations (this is the so called *research team step*). An associated percentage of participation, completed with minor contributors can be useful. If the list is too long, keep updated information in a web page or another document like a SMP, for example, where you can mention the different contributor roles. This is the step in which the intellectual property producer’s rights are established. Producers include the RS authors and rightholders. This is then the step in which RS legal issues related to copyright information are dealt with.
• (*) Establish the list of included software and data components, indicate their licenses (or other documents like the component’s documentation) giving the rights to access, copying, modification and redistribution for each component. In the case of software and data that fall in the category of RS or RD, please take into consideration best citation practices 11, 15.
• Choose a software license, with the agreement of all the rightholders and authors, and establish a signed agreement if possible. The licenses of the software components that have been included and/or modified to produce the RS can have impact in your license decision, see for example 8, 16, 18. Software licenses and licensing information can be found at the Free Software Foundation (FSF) ^5^, the Open Source Initiative (OSI) ^6^, and the Software Package Data Exchange (SPDX) ^7^. Consider using FLOSS licenses to give the rights of use, copy, modification, and/or redistribution. This is then the step in which legal issues related to the RS sharing conditions are to be taken into consideration. Indicate the license in the RS files, its documentation, and the project web pages. Give licenses, like GNU FDL ^8^, Creative Commons (CC) ^9^, LAL ^10^, to documentation and to web sites.
• Choose a web site, forge, or deposit to distribute your product; licensing and/or conditions of use, copy, modification, and/or redistribution should be clearly stated, as well as the best way to cite your work. Good metadata and respect of open standards are always important when giving away new components to a large community: it helps others to reuse your work and increases its longevity. Use Persistent Identifiers (PIDs) ^11^ if possible.
• (*) This step deals with the utility of the RS and how it has been used for your research (this is the *research work step*). Establish the list of main functionalities, and archive a tar.gz or similar for the main RS versions in safe place. Keep a list of the associated research work, including published articles. Update your documentation, SMP, web site, etc. with the new information in each main RS version.
• Inform your laboratories and head institutions about this RS dissemination (if this has not be done in the license step).
• Create and indicate clearly an address of contact.
• Release the RS.
• Inform the community (e.g via mailing lists), consider the publication of a software paper, see for example the list of Journals where you can publish articles focusing on software ^12^.

This proposed procedure is flexible and can be adapted to many different situations.

### 2.3 The CDUR procedure to evaluate Research Software

We include in this section the summarized version of the CDUR protocol that can be found in 11 (section 4.1). This reference gives a detailed description and analysis of the protocol as well as a complete list of references related to this work. This procedure for RS evaluation contains four steps to be applied in the following chronological order: **C**itation, **D**issemination, **U**se and **R**esearch. For example, as we have seen in the Section 2.2, the first steps in the RS dissemination procedure correspond to the correct RS identification, and in order to be correctly cited, the RS reference should be clearly indicated. Let us introduce a resumed version of these four steps.

**(C) Citation.** This step measures to what extent the evaluated RS is well identified as a research output. It is also the step where RS authors are correctly identified as well as their affiliations.

Section 2.5 of 11 proposes three different ways to establish a RS reference, in order to facilitate its citation. Moreover, a more evolved RS identification level could be provided in the form of a metadata set. Reference and metadata include, among other informations, the list of the RS authors and their affiliations ( 11, section 2.2).

**(D) Dissemination.** This step measures the quality of the RS dissemination plan involving actions such as:

• Choosing a license, with the agreement of all the rights’ holders and authors. Consider, preferably, using FLOSS licenses.
• Choosing a web site, forge, or deposit to distribute the product; stating clearly licensing and conditions of use, copy, modification, and/or redistribution.
• Creating and indicating a contact address.

This step deals with legal issues involving the authors and rightholders (as established in the **C**itation step) deciding and installing the license(s) for the RS dissemination. This is also the step concerning Open Science, as the RS license expresses its sharing conditions; and where policy makers should establish the Open Science policies that will be applied in the evaluation process.

Finally, let us recall that the inclusion of the list of related publications, data sets and other related works in the dissemination procedure helps to prepare the reproducible science issues that are to be taken into account in the **U**se step.

**(U) Use.** This step is devoted to the evaluation of the technical software aspects. In particular, this step measures the quality of the RS usage, considering that a performing RS is one that is both correct and usable by the target scientific community.

The RS usability does not only refer to the quality of the scientific output but also can deal with other matters, such as the provided documentation, tutorials and examples (including both inputs and outputs), an easy and intuitive manipulation, testing and version management, etc.

This is the reproducible science step, where it is measured how the published results obtained with the RS can be replicated and reproduced.

**(R) Research.** This step measures the impact of the scientific research that has required in an essential way the RS under consideration.

The evaluation of this item should follow whatever standards for scientific research quality in the concerned community.

This is the step where the RS related publications (as described in the RS definition in Section 2.1) come into play, and where the evaluation should consider the difficulty of the addressed scientific problems, the quality of the obtained results, the efficiency of the proposed algorithms and data structures, etc. The RS impact can also be assessed through the research impact of the related publications, and through its inclusion (or use) as software component in other RS.

Each of these four steps can reach different levels of qualification and the corresponding scale is to be set up by the policy makers considering a particular evaluation event. Thus, the CDUR protocol can be easily adapted to different circumstances: career evolution, recruitment, funding, RS peer review or other procedures to be applied by universities and other research performing institutions, research funders, or scientific journals, and it can also be adapted to different evaluation situations arising in different scientific areas.

### 2.4 FAIR Research Software

Although the FAIR principles have been first designed for data, they apply as well to other digital objects 1:

*…it is our intent that the principles apply not only to ‘data’ in the conventional sense, but also to the algorithms, tools, and workflows that led to that data. All scholarly digital research objects – from data to analytical pipelines – benefit from application of these principles, since all components of the research process must be available to ensure transparency, reproducibility, and reusability.*

In the case of RS, FAIR principles have been considered in several conferences and publications, although some adaptations seem to be necessary 19- 21.

In this section we highlight two points regarding these principles that appear in our RS dissemination procedure (see Section 2.2) and the CDUR evaluation protocol (see Section 2.3), namely those referring to Persistent Identifiers (PIDs) and metadata, as remarked in 5:

*Central to the realization of FAIR are FAIR Digital Objects. These objects could represent data, software, protocols or other research resources. They need to be accompanied by Persistent Identifiers (PIDs) and metadata rich enough to enable them to be reliably found, used and cited.*

Note that these two points are included in the basic “minimum standar” of 5 (p. 13). In particular we would like to observe the following points regarding PIDs:

• we recommend to use PIDs associated to authors, like ORCID ^13^,
• we recommend to associate PIDs to the disseminated RS; as a RS can have several versions, do consider a different PID for each main release,
• as PIDs can be provided by the chosen deposit, PID provision should be one of the arguments favoring the selection of a deposit like, for example, Zenodo ^14^,
• articles associated to the RS should have their own PID, furnishing in this way the RS with other possible citation forms 11 (section 2.5), i.e. with complementary means to reliably finding the RS, facilitating thus its use and citation by other researchers,
• if the included data and software components or other external components that are necessary to run the disseminated RS have associated their own PIDs, it is convenient to refer to them in order to contribute to their own access and visibility.

On the other hand, concerning the role of metadata sets in our RS dissemination and evaluation proposals, let us observe that metadata is a very flexible concept, going from a simple reference or citation form to a very complete and precise RS description. In any case, our protocols consider that they are an important tool to set attribution to the RS and to facilitate credit. One possibility we would like to suggest is the metadata format proposed in the PRESOFT SMP template 10, that has a manageable size and has also the advantage that it is based in the RS index card elaborated in the PLUME project (2006-2013). See, for example, the index card corresponding to OpenMVG ^15^, a RS developed at the Laboratoire d’informatique Gaspard-Monge. A different, more complex metadata set can be generated, for example, with COdeMeta ^16^.

Finally, we consider the implementation and adoption of FAIR principles 1, 5, 7, 22 and other standards as arguments favoring the choice of a deposit for the RS. A tool to help taking such decision could be the FAIRsharing platform ^17^, that provides a large amount of community-developed standards, as well as indicators (among others) necessary to monitor their adoption, and to follow data policies established by funders, editorials and other organizations. See, for example, the information that appears in the FAIRsharing platform associated to the FAIR Principles ^18^.

## 3. Research Data

This section translates to RD the previously addressed RS issues: definition, dissemination and evaluation, ending with some RD FAIR considerations.

### 3.1 A Research Data definition

In coherence with the declared parallelism between RS and RD, we consider here the RD definition proposed in 13.

***Research Data** is a well identified set of data that has been produced (collected, processed, analyzed, shared and disseminated) by a (again, well identified) research team. The data has been collected, processed and analyzed to produce a result published or disseminated in some article or scientific contribution. Each research data encloses a set (of files) that contains the dataset maybe organized as a database, and it can also include other elements as the documentation, specifications, use cases, and any other useful material as provenance information, instrument information, etc. It can include the research software that has been developed to manipulate the dataset (from short scripts to research software of larger size) or give the references to the software that is necessary to manipulate the data (developed or not in an academic context).*

Thus, RD has three main characteristics:

• the goal of the collection and analysis is to do research, that is, to answer a scientific question,
• it has been produced by a research team,
• the RD is involved in the obtention of the results presented in scientific articles (as the most important means for scientific exchange are still articles published in scientific journals).

The identified set of data constitute a database in the case the data are arranged in a systematic or methodical way and is individually accessible by electronic or other means 23- 27. The *sui generis* database rights primarily protects the producer of the database and may prohibit the extraction and/or reuse of all or a substantial part of its content for example 23.

Remark that it is becoming a general practice for research funders to ask for a Data Management Plan (DMP) concerning the data generated in a funded project ^19^ 28- 30. See for example the DMPonline platform of the Digital Curation Center (DCC) as a helpful tool to create, review, and share DMPs that meet institutional and funder requirements ^20^. In particular, French research projects can benefit from DMP OPIDoR ^21^.

### 3.2 A procedure for Research Data dissemination

The following procedure has been adapted to RD from the RS dissemination procedure proposed in Section 2.2. Similarly, steps marked with (*) are to be revisited regularly in each version release, if necessary.

Again, as a general recommendation, it is best practice to consider licensing issues and to keep a DMP from the very first stages of the RD development. The RD license establishes the sharing conditions: it gives rights for access, copy, modification, redistribution of the RD, and it can establish reciprocity clauses that should be respected by the potential RD users. It should be put well into place before releasing the RD.

• Choose a name or title to identify the RD, avoid trademarks and other proprietary names, you can associate date, version number… Consider best practices in file names ^22^.
• (*) Establish the list of the persons that have participate to the RD production, that is, the persons who have collected, processed, analyzed, shared and disseminated the RD; as well as their affiliations (this is the so called *research team step*). If the list is too long, keep updated information in a web page or another document like a DMP, for example, where you can mention the different contributor roles. This is the step in which the producer’s rights are established, if any. Producers include the RD authors (in the case there are intellectual property rights associated to the RD) and the corresponding rightholders. This is then the step in which legal issues related to copyright and ownership information are dealt with 25, 26, 31, 32.
• Data can have associated other legal (or ethical) contexts 13, 27, 32, 33, they can be intimately related to the ongoing research work, consider them with the help of legal experts if necessary.
• (*) Establish the list of included software and data components, indicate their licenses (or other documents like the component’s documentation) giving rights to access, copying, modification and redistribution for the component. In the case of software and data that fall in the category of RS or RD, please take into consideration best citation practices 34- 37.
• Choose a data license, with the agreement of all the producers and rightholders, and establish a signed agreement if possible. The licenses of data components that have been included and/or modified to produce the RD can have impact in your license decision 27. Consider using licenses like the Creative Common licenses (V4.0) ^23^ or the Open Data Commons Licenses ^24^, for example. Other data licenses can be found at SPDX ^25^. This is then the step in which legal issues related to the RD sharing conditions are to be taken into consideration. Indicate the license in the RD files, its documentation, the project web pages, etc. Give licenses, like GNU FDL ^26^, Creative Commons (CC) ^27^, LAL ^28^, to documentation and to web sites.
• Choose a web site, forge, or deposit to distribute your product; licensing and conditions of use, copy, modification, and/or redistribution should be clearly stated, as well as the best way to cite your work. Good metadata and respect of open standards are always important when giving away new components to a large community: it helps others to reuse your work and increases its longevity. Use Persistent Identifiers (PIDs) ^29^ if possible.
• (*) This step deals with the utility of the RD and how it has been used for your research (it is then the *research work step*). Establish the list of the main RD research issues that appear in your work and that can facilitate its reuse. Archive a tar.gz or similar for the main RD versions in safe place. Keep a list of the associated research work, including published articles. Update your documentation, DMP, web site… with the new information in each main release.
• Inform your laboratories and head institutions about this RD dissemination (if this has not be done in the license step).
• Create and indicate clearly an address of contact.
• Release the RD.
• Inform the community (e.g. via mailing lists), consider the publication of a data paper.

This proposed procedure is also flexible and can be adapted to many different situations.

A much more complete and complex vision of data sharing can be found, for example, in 5.

### 3.3 The CDUR procedure to evaluate Research Data

Similarly to the RS CDUR evaluation protocol proposed in Section 2.3, the CDUR protocol for RD evaluation that we propose out in here contains four steps to be carried out in the following chronological order: **C**itation, **D**issemination, **U**se and **R**esearch. The RS CDUR protocol translates to RD evaluation in a straightforward way:

**(C) Citation.** This step measures to what extent the evaluated RD is well identified as a research output. It is also the step where RD producers are correctly identified as well.

As seen in the dissemination procedure ( Section 3.2), a reference to cite the work should be well established. If required in a evaluation process, a complete set of RD metadata should be provided.

**(D) Dissemination.** This step measures the quality of the RD dissemination plan, as seen in the previous Section 3.2.

This is also the step dealing with legal (and ethical) issues 13, 27, 32, 33 related to the producers and rightholders (as established in the **C**itation step) deciding and installing the license(s) for the RD dissemination. It can also take into consideration further legal issues related to the objects under study represented in the RD and their legal contexts ( 13, section 3).

This is also the step concerning Open Science, as the RD license expresses its sharing conditions; and the step where policy makers should establish the Open Science policies that will be applied in the evaluation process.

Finally, let us recall that the inclusion of the list of related publications, software and data sets and other works mentioned in the dissemination procedure helps to prepare the reproducible science issues that are to be taken into account in the **U**se step.

**(U) Use.** This step is devoted to the evaluation of the technical data aspects. In particular, this step measures the quality of the RD. The RD usability does not only refer to the quality of the scientific output but also can deal with other matters, such as the provided documentation, tutorials and examples of use for easy and intuitive manipulation, etc.

This is the reproducible science step, where it is measured how the published results obtained with the RD can be replicated and reproduced.

**(R) Research.** This step measures the impact of the scientific research that has required in an essential way the RD under consideration.

The evaluation of this item should follow whatever standards for scientific research quality in the concerned community.

This is the step where the RD related publications (as described in Section 3.1) come into play, and where the evaluation should consider the difficulty of the addressed scientific problems, the quality of the obtained results, the efficiency of the proposed algorithms and data structures, etc. The RD impact can also be assessed through the research impact of the related publications, and through its inclusion (or use) as a data component in other RD.

To end this section, let us remark that similar considerations for the flexibility of the application of the CDUR RS evaluation protocol do apply for RD.

### 3.4 FAIR Research Data

Remark that, as stated in Section 2.4, FAIR principles have been initially designed for data, so there is no need for a more detailed description here. Indeed, there is a lot of recent work on FAIR data issues, see for example 1, 5, 7, 22, 38 and the references mentioned there.

As we have seen all along in Section 3, what we have proposed for RS translates directly to RD: definition, dissemination, evaluation. Therefore, we think that the same applies for the points studied in the Section 2.4, namely PIDs and metadata: they find as well a direct translation to RD, so we think that there is no real need to develop them in here again.

Finally, to complete our considerations on FAIR issues, we would like to mention some FAIR assessement tools currently under development, such as the automatic FAIR evaluator (DIGITAL.CSIC) of the EOSC-Synergy project ^30^ or the data sharing evaluation project ^31^.

## 4. Conclusion

Designing and following best practices for research output dissemination are important steps toward accomplishing the Open Science goals, to render research visible, accessible and reusable 2. We also consider that the current evolution in research evaluation practices will enable the adoption of Open Science methods 39, 11, as well as they will facilitate their integration in every day research activities.

As we have already detailed in our work, RS and RD present many similarities concerning dissemination and evaluation issues. For example, we have included in Section 3.1 a RD definition that has been proposed in 13 and that it is clearly based on a RS definition (see 11, 12 and Section 2.1). Following the same scheme, in Section 3 we have proposed and argued in detail RD dissemination and evaluation procedures grounded in the RS proposed dissemination ( Section 2.2 and 8) and evaluation ( Section 2.3 and 11) procedures.

It is pending work for the future to analyse the potential extension of this parallelism to other kinds of research outputs that are disseminated under similar conditions as RD and RS, that is, without widely accepted publication procedures involving editors or other external actors and where the dissemination is usually restricted through the hands of the production team (eventually including platforms or repositories).

Sections 2.4 and 3.4 on FAIR RS and RD issues study the role of PIDs and metadata in the proposed dissemination and evaluation protocols. Yet, we can notice that the FAIR principles do not provide precise dissemination and evaluation provisions but general guidelines 1, 7. We consider that our dissemination and evaluation (CDUR) proposals, if followed correctly, may clearly contribute towards a more sound implementation of FAIR principles.

In fact, our proposals here provide concrete instructions for RD and RS producers to make them more findable and accessible, as well as arguments to choose suitable dissemination platforms to complete the FAIR framework. Moreover, interoperability and reusability could be also fostered with best documentation practices, such as it is proposed in our dissemination procedure; practices that can be evaluated with our CDUR protocol. Furthermore, we consider that our dissemination and evaluation mechanims contribute towards open access outputs as we highlight precisely the steps that deal with licensing issues.

On another note, we consider that one of the advantages of the CDUR protocols for RS and RD described here is that they separate the evaluation of research aspects from those related to much more technical issues concerning software or data, as these different contexts may involve evaluators with disparate levels of expertise in the corresponding areas.

As remarked in Sections 2.3 and 3.3, the first two steps of the CDUR protocols correspond to best dissemination practices. A research output that is to be disseminated should be identified correctly to increase the visibility of the output, as well as the visibility of its producer team and their research work, in order to make it accessible and reutilizable. We have already highlighted in 11 that one of the roles of the evaluation stages is to improve best dissemination practices, such as best credit, attribution and citation, practices that are still to be widely adopted:

*… we consider that it is in the interest of the research communities and institutions to adopt clear and transparent procedures for the evaluation of research software. Procedures like the proposed CDUR protocol facilitate RS evaluation and will, as a consequence, improve RS sharing and dissemination, RS citation practices and, thus, RS impact assessment.*

Finally, we would like to emphasize the dissemination/evaluation loop: first, the CDUR protocol points out to the research community the need to correctly disseminate outputs, as only well disseminated outputs are potential subject of evaluation; secondly, the CDUR protocol also implies that outputs are to be disseminated following the adopted evaluation policies.

In this imbricated context, it is the intention of this work to contribute towards improving dissemination and evaluation procedures, and thus, to enhance best Open Science every day practices.

## Data availability

### Underlying data

Data underlying the arguments presented in this article can be found in the references and footnotes.

## Footnotes

^1^

https://en.wikipedia.org/wiki/Free_and_open-source_software

^2^

*Diffuser un logiciel de laboratoire: recommandations juridiques et administratives*, 2010, https://projet-plume.org/fr/ressource/diffuser-logiciel-recomm-juridiques-admin. In French.

^3^

https://projet-plume.org/, https://projet-plume.org/en

^4^

See for example https://libguides.princeton.edu/c.php?g=102546&p=930626, https://doranum.fr/stockage-archivage/comment-nommer-fichiers/

^5^

https://www.fsf.org/licensing/

^6^

https://opensource.org/licenses

^7^

https://spdx.org/licenses/

^8^

http://www.gnu.org/copyleft/fdl.html

^9^

https://creativecommons.org/choose/

^10^

http://artlibre.org/licence/lal/en/

^11^

http://en.wikipedia.org/wiki/Persistent_identifier

^12^
This list is mantained by Neil Chue Hong in the Software Sustainability Institute web page https://www.software.ac.uk/which-journals-should-i-publish-my-software

^13^

https://orcid.org/

^14^

https://zenodo.org

^15^

https://projet-plume.org/en/relier/openmvg

^16^

https://codemeta.github.io/codemeta-generator/

^17^

https://fairsharing.org/

^18^

https://fairsharing.org/FAIRsharing.WWI10U

^19^

https://ec.europa.eu/research/participants/docs/h2020-funding-guide/cross-cutting-issues/open-access-data-management/data-management_en.htm

^20^

https://dmponline.dcc.ac.uk/

^21^

https://opidor.fr/planifier/

^22^

See for example https://libguides.princeton.edu/c.php?g=102546&p=930626, https://doranum.fr/stockage-archivage/comment-nommer-fichiers/

^23^

https://creativecommons.org/choose/

^24^

https://opendatacommons.org/licenses/

^25^

https://spdx.org/licenses/

^26^

http://www.gnu.org/copyleft/fdl.html

^27^

https://creativecommons.org/choose/

^28^

http://artlibre.org/licence/lal/en/LAL

^29^

http://en.wikipedia.org/wiki/Persistent_identifier

^30^

https://github.com/EOSC-synergy/FAIR_eva

^31^

https://hal.archives-ouvertes.fr/hal-01943521
