# Supplementary material for: Research Software vs. Research Data II: Protocols for Research Data dissemination and evaluation in the Open Science context
Source: F1000Res. 2022 Oct 7;11:117. Originally published 2022 Jan 28. [Version 2] doi: 10.12688/f1000research.78459.2 (PMC9706143; doi:10.12688/f1000research.78459.2)
Supplement: Peer review history

# Peer review: Reviewer response for version 2

**DOI:** 10.5256/f1000research.138948.r152672

**Published:** 2022-11-28

**Authors:** Charles Romain, Henry S. Rzepa

**Affiliations:** 1 Department of Chemistry, Molecular Sciences Research Hub, Imperial College London, London, UK

The authors addressed the various comments and recommendations by adding text or table. We recommend for indexing.

Is the work clearly and accurately presented and does it cite the current literature?

Yes

If applicable, is the statistical analysis and its interpretation appropriate?

Not applicable

Are all the source data underlying the results available to ensure full reproducibility?

No source data required

Is the study design appropriate and is the work technically sound?

Yes

Are the conclusions drawn adequately supported by the results?

Partly

Are sufficient details of methods and analysis provided to allow replication by others?

Yes

Reviewer Expertise:

FAIR data, workflows for data publication, chemistry

We confirm that we have read this submission and believe that we have an appropriate level of expertise to confirm that it is of an acceptable scientific standard.

# Peer review: Reviewer response for version 2

**DOI:** 10.5256/f1000research.138948.r152673

**Published:** 2022-10-07

**Authors:** Mark Leggott

**Affiliations:** 1 Digital Research Alliance of Canada, Ottawa, ON, Canada

I feel the authors have addressed my comments, either via edits, or explaining why they feel the specific comment does not apply (e.g. SBOMs).

Is the work clearly and accurately presented and does it cite the current literature?

Partly

If applicable, is the statistical analysis and its interpretation appropriate?

Not applicable

Are all the source data underlying the results available to ensure full reproducibility?

No source data required

Is the study design appropriate and is the work technically sound?

Partly

Are the conclusions drawn adequately supported by the results?

Partly

Are sufficient details of methods and analysis provided to allow replication by others?

Yes

Reviewer Expertise:

Research data management, research software, digital research infrastructure.

I confirm that I have read this submission and believe that I have an appropriate level of expertise to confirm that it is of an acceptable scientific standard.

# Peer review: Reviewer response for version 1

**DOI:** 10.5256/f1000research.82451.r125654

**Published:** 2022-04-26

**Authors:** Mark Leggott

**Affiliations:** 1 Digital Research Alliance of Canada, Ottawa, ON, Canada

In general I found the intent of the article (to propose a common rubric for making data and SW adhere to Open Science and FAIR Principles) to be a reasonable goal, but I'm not sure the article clearly achieves that goal. The authors state that "Bearing in mind the above described landscape, the goal of our work here is to contribute to the improvement of the scientific endeavor with protocols that could help researchers, and the community at large, in the dissemination of their produced RD and RS, while contributing to the accomplishment of Open Science goals." I don't feel that the proposed protocols (CDUR) provide sufficiently detailed recommendations to support this goal.

 Part of the challenge I have in saying this is that work of initiatives like FAIR4RS would be very informative in this article, but they are not mentioned. Also, the FAIR4RS Principles have direct intersections with some of the CDUR approaches, which seems overly simplified in view of this FAIR4RS rubric. I find it unusual that the paper does not even mention the work of the FAIR4RS Working Group, which has articulated a number of the concepts and approaches highlighted in this article. The work of the RDA-FORCE11 Software Source Code Identification Working Group is also of direct relevance to the PID discussion in 2.4, so should be highlighted.

 A table comparing the CDUR recommendations against data and SW might be useful, as it would extract the key elements of the proposed approach and make it easier for the reader to make the connections.

 The authors could mention the value of a Software Bill of Materials (SBOMs) in section 2.1, para 4 (Moreover...) Mentioning how a SMP can be integrated with a DMP, reinforcing the idea that the data and SW can be considered in a common rubric, would also be beneficial. There has been some work by the Software Sustainability Institute to develop a SMP meant to be integrated with standard DMP tools.

 I find the idea of drawing connections between the practices needed to support similar Open Science/FAIR concepts with data and SW is very desirable, and the authors do provide one of the few attempts to articulate this. If they were able to achieve a better integration of additional and specific resources and best practices with their CDUR approach, it would benefit the article substantially.

Is the work clearly and accurately presented and does it cite the current literature?

Partly

If applicable, is the statistical analysis and its interpretation appropriate?

Not applicable

Are all the source data underlying the results available to ensure full reproducibility?

No source data required

Is the study design appropriate and is the work technically sound?

Partly

Are the conclusions drawn adequately supported by the results?

Partly

Are sufficient details of methods and analysis provided to allow replication by others?

Yes

Reviewer Expertise:

Research data management, research software, digital research infrastructure.

I confirm that I have read this submission and believe that I have an appropriate level of expertise to confirm that it is of an acceptable scientific standard, however I have significant reservations, as outlined above.

# Response

**Published:** 2022-09-08

**Authors:** Teresa Gomez-Diaz

**Affiliations:** LIGM, Gustave Eiffel University & CNRS, France

Dear Mark Leggott,

 Many thanks for all your comments that have helped us a lot to improve our work. Here are some answers while we are preparing a new version.

 - [intent of the article]

 - [proposed protocols (CDUR) provide sufficiently detailed recommendations]

 Of course, it is our opinion that the article achieves that goal to a sufficient extent as to merit its approval by the Referees.

 - [initiatives like FAIR4RS...]

 Perhaps you have not noticed that in the original version of this paper there were specific references to FAIR RS in References 19,20,21. In the new version we have added more recent ones following your suggestion. We will also include Research Data Alliance/FORCE11 Software Source Code Identification WG, Allen, Alice, Bandrowski, Anita, et al. (2020). Software Source Code Identification Use cases and identifier schemes for persistent software source code identification (1.1). https://doi.org/10.15497/RDA00053. Many thanks for this suggestion.

 - [table table comparing the CDUR recommendations against data and SW]

 Thanks for your suggestion, but we do not think a comparison table is necessary, as CDUR for RS and for RD find pretty similar formulation, only a new item for RD dissemination (item 3 in Section 3.2) has been added to the RS dissemination protocol to highlight the potential difficulties concerning RD legal (and ethical) issues.

 - [Software Bill of Materials (SBOMs)]

 Many thanks for your suggestion, but we consider that software security issues, the main topic of, for example, the report https://linuxfoundation.org/wp-content/uploads/LFResearch_SBOM_Report_020422.pdf, are not part of our conceptual approach of Section 2.1. As already mentioned to other Referees, we do not enter in several concrete and technical issues. The point of software security issues, as well as many other ones, could be considered by the evaluation committees in the Use step of the CDUR protocol, which can be easily adapted by the committees to consider such technical points.

 Indeed, there are many relevant issues that are not detailed in our definition and proposed protocols, as we aim to address the subject from a more conceptual perspective. This point of view has been clarified in the new version of [Reference 13]. The decision of considering such broad point of view has required us to include in the new version of [Reference 13] comments reacting to some Referee questions that asked us, like you do, the consideration of different specific issues that they considered we had forgotten.

 - [how a SMP can be integrated with a DMP]

 Thanks again for your suggestion. This could be the subject of future extension of our work, but it is out of the scope of the present article.

 - [I find the idea of drawing connections between the practices needed to support similar Open Science/FAIR concepts with data and SW is very desirable, and the authors do provide one of the few attempts to articulate this.]

 Thank you so much for this very positive comment, that in some sense supports our perception that this article already provides enough contributions to merit its publication as it is, although, there is always room for improvements in future work.

 - [achieve a better integration of additional and specific resources and best practices]

 Following your suggestion we provide a new version of sections 2.4 and 3.4 regarding additional reflections on the relationships between FAIR and CDUR issues. For more detailed information on the CDUR protocol the appropriate reference is [Reference 11], while in the present paper we have included only a short description to explain that CDUR can be applied in a similar way for RS and for RD.

 Teresa Gomez-Diaz, Tomas Recio

# Peer review: Reviewer response for version 1

**DOI:** 10.5256/f1000research.82451.r121544

**Published:** 2022-02-07

**Authors:** Charles Romain, Henry S. Rzepa

**Affiliations:** 1 Department of Chemistry, Molecular Sciences Research Hub, Imperial College London, London, UK

This manuscript aims to highlight similarities between research software (RS) and research data (RD) in the context of Open Science. The authors propose protocols and procedures for the dissemination and its evaluation for both RS and RD. The introduction provides a clear overview of the context by citing relevant sources. The manuscript then discusses definition, dissemination procedures and evaluation protocols for both RS and RD.

 The comparison of RS and RD in the context of dissemination (and its evaluation) is interesting and highlights some similarities in the bottlenecks and challenges that need to be addressed.

 However, our main general concern is the high heterogeneity of RD compared to RS (maybe a naïve vision) which makes these general procedures and protocols not always applicable or too vague to be practically useful as they don’t provide practical solutions.

 However, they have the merit to draw the attention to the importance of following good practices (e.g. protocols, procedures) to disseminate RD and RS, and provide general guidance which can help to identify what could be some potential practical solutions. We recommend this article to be indexed as a good basis for further discussions.

 Below we provide some detailed comments and suggestions on specific points discussed in the manuscript that could be included in future version of the article.

**Proposed definitions:**

- The proposed definitions used in this article are based on another article currently awaiting peer review ^1^. The authors should update these definitions after comments from the reviewers and approval of the other manuscript.
- We generally agree with the proposed definitions. However, regarding the definition of RD we would add that following protocols and standards established in the field are important. For example, “ *the data has been collected, processed and analysed **following protocols, procedures and standards** established in the field to produce a results…..”*

**Dissemination and evaluation procedures:**

- The dissemination procedure and the CDUR protocol for RS have previously been reported and reviewed, we don’t have further comments on these.
- One important aspect and challenge for RD dissemination that should be mentioned is to make data “machine-readable” to facilitate collection and enable re-use, and thus contribute to new data-driven discoveries (e.g. using machine-based tool). In some fields, RD are mainly shared as “human-readable” format only, usually in a monolithic supporting information document along with a scientific publication (e.g. PDF format). RS in contrast has well developed repositories which to a certain extent are machine actionable, as well as being closely integrated into the publication processes (such as Github via https://github.com/openjournals and https://joss.theoj.org). Overall, we think this article should have a paragraph where the state of play might be on machine-readable or even machine-actionable RD and RS and perhaps comparing how this is evolving for both of them.
- In general, it would be good to further emphasise that many aspects discussed in these procedures and protocols can easily be addressed with relevant metadata which are keys to address the FAIR principles. In terms of metadata, this plays a key role in ensuring Findability/Discovery of the object, using the metadata registry MDS (metadata store). It would be good for this article to perhaps illustrate the role of registered metadata in finding both RD and RS, and perhaps to explore the granularity of the metadata for both. Is it good enough to use metadata purely to discover the functionality of a RS code, or might it be necessary to explore in more details the functions and libraries in RS?
- It is maybe a naïve vision, but research data features a much broader heterogeneity than RS, so many different types of data can be generated that it makes these procedures difficult to apply or generalise to RD in general (As mentioned in the report of reviewer 1 for RS ^2^).
- In general, more specific examples are provided for RS than for RD. More examples or references for RD would be useful for the readers.

*Dissemination steps:*

- Contrary to RS, versioning is rare or can be difficult to achieve with RD. Complementary data can be generated to support new conclusions but does not necessarily feature new versions of the previous ones, thus “ *revisiting version release”* as mentioned doesn’t necessarily make sense, but *each time new data are generated* would be more appropriate.
- A single name or title for RD is not necessarily as straightforward as it is with RS, as RD usually are made available along with scientific articles (as mentioned in the three main characteristics), the name of the dataset can be related to the title of the narrative it accompany though.
- *"Choose a web site, forge…"* Data repository should be explicitly mentioned. In addition, selecting data repository which generate relevant metadata for the discipline (if available) should be considered (rather than generic data repository).
- The address of contact can easily be addressed via PID associated with the authors (i.e. ORCiD).

*Evaluation steps:*

- We would encourage the author to discuss how the different C, D, U, R steps of the protocol help to meet the FAIR criteria. To some extent, the Citation step help to address the Findability, the Use step deals with the interoperability, etc…

*Data citation:*

- These can easily be evaluated via the attribution of DOI, and PID for the researchers (OrCiD). The data citation can follow some guideline previously defined by various organisation (see joint declaration for data citation - FORCE11) and should be given as examples.

The choice of file format (e.g. non-proprietary, open format, machine-readable) to enable re-use is important and something that should be evaluated in the protocol.

 The replicability and reuse steps are highly challenging for RD, even if RD are “well-disseminated”. We would emphasize the importance of the context and how the RD have been generated, to maximize reuse and reproducibility.

Is the work clearly and accurately presented and does it cite the current literature?

Yes

If applicable, is the statistical analysis and its interpretation appropriate?

Not applicable

Are all the source data underlying the results available to ensure full reproducibility?

No source data required

Is the study design appropriate and is the work technically sound?

Yes

Are the conclusions drawn adequately supported by the results?

Partly

Are sufficient details of methods and analysis provided to allow replication by others?

Yes

Reviewer Expertise:

FAIR data, workflows for data publication, chemistry

We confirm that we have read this submission and believe that we have an appropriate level of expertise to confirm that it is of an acceptable scientific standard, however we have significant reservations, as outlined above.

## References

[1] : Research Software vs. Research Data I: Towards a Research Data definition in the Open Science context. *F1000Research* .2022;11: 10.12688/f1000research.78195.1 10.12688/f1000research.78195.1 36415208PMC9650106

[2] : On the evaluation of research software: the CDUR procedure. *F1000Research* .2019;8: 10.12688/f1000research.19994.1 10.12688/f1000research.19994.1 PMC688339931814965

# Response

**Published:** 2022-09-08

**Authors:** Teresa Gomez-Diaz

**Affiliations:** LIGM, Gustave Eiffel University & CNRS, France

Dear Charles Romain and Henry S. Rzepa,

 Many thanks for all your comments that have helped us a lot to improve our work. Here are some answers while we are preparing a new version.

 - [high heterogeneity of RD compared to RS]

 We will include some explanations. Indeed, heterogeneity appears obviously between software and data, yes. But if we keep these aspects aside and focus on the similarities, we can learn a lot from the common features that appear in the production context of RD and RS. This is a general procedure that can be adapted to several situations. Even when the differences are too important, and maybe the proposed dissemination and evaluation procedures are not directly applicable as such in both settings, they could help to suggest hints to address the diverse issues appearing in each environment.

 - [to draw the attention to the importance of following good practices]

 Many thanks!

 - [Proposed definitions, article currently awaiting peer review]

 We are preparing Version 2 of [Reference 13] bearing in mind Referees’ comments.

 - [Proposed definitions, would add following protocols and standards]

 We agree with you, but we do not think that is necessary to change the formulation of our definition. Yet, in the new version of [Reference 13], we have detailed and emphasized the

 relevance, in order to characterize the RD concept, of the research team decisions in the scientific production framework, which includes the *protocols, procedures and standards to be followed* in order to consider some given data as true RD. See, for example, our reference to the STRENDA standards developed for investigations on enzyme activities. Some of your comments to the present paper have helped us to improve the new version of [Reference 13], as they are related to the conceptual framework. For this reason, the new version of the present paper does not reflect them in here.

 - [Dissemination and evaluation procedures, machine-readable...]

 Yes, there are many relevant issues that are not detailed in our definition and proposed protocols, as we aim to address the subject from a more conceptual perspective. This point of view has been clarified in the new version of [Reference 13]. The decision of considering such broad point of view has required us to include in the new version of [Reference 13] comments reacting to some Referee questions that asked us, like you do, the consideration of different specific issues that they considered we had forgotten.

 - [Dissemination and evaluation procedures, a paragraph where the state of play might be on machine-readable or even machine-actionable RD and RS...]

 Again, out of our scope/goals, we agree that machine actionable is an important issue, but we do not enter in this kind of very concrete aspects.

 Some of your comments to the present paper have helped us to improve the new version of [Reference 13], as they are related to the conceptual framework. For this reason, the new

 version of the present paper does not reflect them in here.

 - [Dissemination and evaluation procedures, FAIR principles....]

 We deal with FAIR issues Sections 2.4 and 3.4, and as remarked there, we do remain in a conceptual level that does not enter in many challenging issues like the ones you mention.

 - [Dissemination and evaluation procedures, research data features a much broader heterogeneity than RS]

 See our comment above about heterogeneity issues.

 - [Dissemination and evaluation procedures, more specific examples are provided for RS than for RD]

 It seems to us that the targeted research audience is used to handle RS and/or RD as part of their everyday research practices, so they do not require further examples. Anyway, we have made references to different aspects of linguistic, environmental and geographical data in Section 3 of [Reference 13]. Moreover, further examples can be found easily in the literature, as can be seen in the bibliography included at the end of both works (the present article and [Reference 13]) .

 - [Dissemination steps, RD versioning is rare]

 References 49 and 50 of [Reference 13] do mention data versioning as the standard term. Indeed there are cases where versioning could be a difficult issue. But we consider that the RD production team can select the data that is to be released, and maybe provide several versions of the same RD object, thus, “version” seems to be the correct term. The inclusion of the sentence “each time new data are generated” could generate confusion: are we are referring to the same RD object or not? This is similar to what happens to new versions or main releases of software.

 - [Dissemination steps, single name or title for RD is not necessarily as straightforward]

 When we speak about the publications associated to RS or RD this means the place where the obtained results are published. Several articles or other scientific contributions (e.g. conferences, books…) can present the scientific results obtained with the RS and/or the RD. These objects (articles, RS, RD) can have different names. See for example the list of publications related to SageMath-Combinat [ https://www.sagemath.org/library-publications-combinat.html] or the RD entitled “Vital Statistics data” associated to the publication [Recio Alcaide A, Pérez López C, Bolúmar F. Influence of sociodemographic factors in birth seasonality in Spain. Am J Hum Biol. 2022 Aug 8:e23788. doi: 10.1002/ajhb.23788].

 It is usually difficult to foresee if some lines of code, or some collected data will become few months later more organized RS or RD. The fact of giving a name to this initial object is the first step to identify it as a research output.

 - [Dissemination steps, Data repository should be explicitly mentioned]

 Yes, you are right. This point has been studied in the Conclusions of [Reference 13], that is the Where? question of the RD Borgman’s conundrum challenges. We will modify in the new version of the present article the RD dissemination procedure to include your suggestion.

 - [Dissemination steps, The address of contact can easily be addressed via PID associated with the authors (i.e. ORCiD).]

 Well, we do not fully agree with this optimistic view, as ORCID usually refers to one researcher, while a mail address can refer to a (evolving) team, which is a much more generic solution. In our opinion, the identification issue can still be a quite challenging issue, mainly for software or data that have been developed for many years collectively. Thus, we do prefer to keep the current formulation in Sections 2.2 and 3.2 of the present article.

 - [Evaluation steps, how the different C, D, U, R steps of the protocol help to meet the FAIR criteria]

 Yes, you are right, and many thanks for this interesting suggestion, that have addressed in sections 2.4 and 3.4 of the new version.

 - [Data citation, can easily be evaluated via the attribution of DOI..]

 Citation examples are already available in the cited works, see for example References 4,34,35,37, and we will add this one: Altman M, Crosas M. The evolution of data citation:

 From principles to implementation . IASSIST Quarterly. 2013;37]. See also our comments above concerning ORCID issues.

 - [choice of file format]

 Yes, but we think that this issue is already addressed – perhaps not in such level of detail as you mention, as this level does not correspond to the more conceptual approach of our work – , as indicated in the dissemination protocols, in Sections 2.2 and 2.3:

*Good metadata and respect of open standards are always important when giving away new components to a large community: it helps others to reuse your work and increases its longevity.*

 And this is to be evaluated in the CDUR protocol in the Dissemination step, as the use of open formats corresponds to Open Science issues. Other technical issues can be evaluated in the Use step, see the description of this step in section 4.2 of [Reference 11].

 - [The replicability and reuse steps are highly challenging for RD, even if RD are “well-disseminated”. We would emphasize the importance of the context and how the RD have been generated, to maximize reuse and reproducibility.]

 Yes, see [Reference 13] where the importance of the context, that is intimately related to the data and RD concepts, is examined thoroughly in Section 3 (OECD Glossary of Statistical Terms and Reference 18 indicated in  [Reference 13]. Following your suggestion, this point has been highlighted now in the new version in preparation, see the CDUR protocols proposed for RD (Section 3.3).

 Teresa Gomez-Diaz, Tomas Recio
